# Supplementary material for: The development of respect in young athletes: A systematic review and meta-analysis
Source: PLoS One. 2021 Jun 3;16(6):e0252643. doi: 10.1371/journal.pone.0252643 (PMC8174680; doi:10.1371/journal.pone.0252643)
Supplement: Attachment — Submitted filename: General comments to the authors.docx [file pone.0252643.s002.docx]

**The development of respect in young athletes: A systematic review and meta-analysis**

First of all, the reviewer would like to thank the authors for their work and efforts in trying to improve sports science knowledge.

**General comments to the authors**

Overall, this is a nice study that could have well systematic evaluation when integrated with the practice of sports among young people relating he development of values. The authors are commended on their efforts thus far. The study is well well-written and a great systematic review and meta-analysis. However, I suggest only small corrections to the authors and these corrections will allow improving the manuscript.

**Abstract**

Line 27: total effect size should be written with its’ magnitudes

Line 29: total effect size should be written with its’ magnitudes

**Introduction section**

Line 65: the authors should delete (p. 15)

**Methods** **section**

Line 118: the authors should rewrite sentence especially dates

Line 186: the authors should be attention to write capital letter throughout the article

Line 270: the authors should delete (p. 404)

**Results section**

This section is well designed and well-written.

**Discussion section**

Overall the discussion is well-written and incorporates relevant literature.

Overall the discussion section should be re-design according to journal guidelines especially margins

**References**

The authors should check writing style of references according to journal guidelines
