# Supplementary material for: The development of respect in young athletes: A systematic review and meta-analysis
Source: PLoS One. 2021 Jun 3;16(6):e0252643. doi: 10.1371/journal.pone.0252643 (PMC8174680; doi:10.1371/journal.pone.0252643)
Supplement: Attachment — Submitted filename: Response to Reviewers.docx [file pone.0252643.s003.docx]

**REBUTTAL LETTER**

Manuscript PONE-D-21-05952 [EMID:0e2927bc8a1101f0]

The development of respect in young athletes: A systematic review and meta-analysis

PLOS ONE

| **Reviewer 1’s comments and suggestions for authors** | **Details of the revisions and responses** |
| --- | --- |
| Abstract  Line 27: total effect size should be written with its’ magnitudes  Line 29: total effect size should be written with its’ magnitudes | We have added the magnitude of the total effect size |
| Introduction section  Line 65: the authors should delete (p. 15) | We have deleted (p. 15). |
| Methods section  Line 118: the authors should rewrite sentence especially dates  Line 186: the authors should be attention to write capital letter throughout the article  Line 270: the authors should delete (p. 404) | We have rewritten the sentence concerning the dates  We have paid attention to capitalization throughout the article  We have deleted (p. 404) |
| References  The authors should check writing style of references according to journal guidelines | We have checked the references according to journal guidelines |

**THANK YOU** for your comments and suggestions

| **Reviewer 2’s comments and suggestions for authors** | **Details of the revisions and responses** |
| --- | --- |
| Introduction  I think that a phrase regarding the novelty of the study would be necessary in this chapter to be introduced | We have introduced a sentence about this question:  As far as we know, the study of the effects of interventions focused on the development of respect is novel. For this reason, and considering the importance… |
| Line 118 - ''articles published between 2000 16 and 2020'', I think it's a mistake of word processing, please correct it....is 2000 - 2020, or 2000, 2016, 2020??? | We have rewritten the sentence concerning the dates |
| Another question for the authors refers to the fact that it has been highlighted that most of the studies are from Spain. So, do you have articles from other countries or did you want to analyze especially the research in this country? it is quite strange that 5 articles are from Spain and only one from another country, please detail because this aspect is important and I think that this choice should be highlighted in the article, as clearly as possible | Regarding this question, we must stress that the search, and the subsequent analysis, did not focus on studies conducted exclusively in Spain, but these six articles were the ones that met the inclusion criteria after the search carried out in the different databases.  In this sense, we have introduced de following sentence:  It should be highlighted that, although five of the six researches had been carried out in Spain, the intention of the researchers was to include the studies carried out in any country |
| Why in Figure 1 the authors state that ''Studies included in qualitative and quantitative synthesis (meta-analyses) (n = 5/7)''? .......there are not 6 articles analyzed in the end, the figure shows that there are 5 or 7, please explain | This is because for the quantitative analysis of the respect to opponents and to the rules and referees, they were analyzed by 5 studies. In addition, seven studies were reviewed with regard to respect for social conventions (see Figures 2-4). It should be remembered that one of the manuscripts included two experimental groups |

**THANK YOU** for your comments and suggestions

| **Reviewer 3’s comments and suggestions for authors** | **Details of the revisions and responses** |
| --- | --- |
| The background of the paper (Line 11-15) is about sport and the promotion of respect in the practice of sport among young people, but later in the paper, we can see that authors mention teachers (Line 40, line 45...) and physical education classes, so it is necessary to harmonize the terminology | In this sense, the authors of this paper consider that the practice of sports performed by young athletes can be developed, both in physical education classes, in which sports are often a subject frequently used by physical education teachers, and in sports clubs, and that in both contexts it is important to promote respect. Here we are talking about young people who practice sports, regardless of the context |
| We can see that there are six types of research included in the quantitative analysis, four were performed in Secondary Education with boys and girls between 12 and 18 years old, one in Primary Education (10-12 years old), and only one in a football club (10-12 years old). Authors cannot state that “the development of respect in the context of sport can be successfully achieved through a planned and deliberate intervention” based on only one study | We have modified this statement:  Based on the study conducted, it can be concluded that the development of respect among young athletes can be successfully achieved through a planned and deliberate intervention, mainly in physical education classes |
| The teacher and the coach have different goals so we cannot look at these problems together in the context of sports and physical education, so (Line 68-70) refers to sportsmanship, which does not have to be relevant for physical education classes at school | We agree with the reviewer when he says that the goals of the teacher and the coach are different. However, we believe that in teaching games, and with young people, both should have similar objectives in terms of education. For this reason, we believe that sportsmanship is relevant in both contexts |
| Line 253-261 repetition from the method section | As stated by the reviewer, these lines mention the databases again, but provide new information: documents selected from each of the databases, additional studies found, and manuscripts excluded according to the inclusion criteria and studies included in the research carried out. This information complements that provided by Figure 1, and we think it’s important |
| Line 269: substantially_different | We have corrected this issue |
| Line 173-238 is part of the data collection but consisted of the methodology of the interventions in earlier studies. It is not very clear what is the goal of this section. It is a description of the papers and it is too long. We can see that the authors used a different methodology | The aim of this part is to describe the most important characteristics of the interventions made in the analyzed manuscripts. However, its length has been shortened as suggested by the reviewer |

**THANK YOU** for your comments and suggestions

| **Reviewer 4’s comments and suggestions for authors** | **Details of the revisions and responses** |
| --- | --- |
| The rationale of the present study should be further highlighted. Is the development of personal and social responsibility over the life span important? Of course, it is. Is the respect an important value related to the implementation of good behaviors in sport? Yes, the sport context follows this value and assumes it as a “rule” in and out of the field inclusively. So, could the authors further highlight the pertinence of the study? | The authors have underlined the relevance of the study:  In this way, children who participate in sport can benefit from the development of a range of personal and social skills, such as skills for relationships with peers and for personal and social responsibility, as well as pro-social behaviours such as respect. Therefore, it is pertinent to study the development of respect in young people through sport practice |
| In the method’ section, a concern of the present study was that the search strategy. Using the current keywords may not be able to include all the related papers. The authors may need some references to support the usage of the current keywords. According to the Figure 1, the initial search only included around 500 papers covering such a broad topic. Therefore, references are necessary to support these keywords. Why not include papers published before 2000? Why did the authors limit the filter of search to three languages? | Dear reviewer, we think your reflections are very interesting. In this regard, it should be mentioned that for the selection and inclusion of the keywords of the search carried out, numerous documents on the subject under study were consulted, as well as the keywords of those documents. Mention should also be made of the fact that the criteria for inclusion were very demanding. This was because the goal was to obtain research that had control and experimental groups, as well as pre-test and post-test reporting results through means and standard deviations. All this in order to be able to carry out the meta-analyses  With regard to the criterion of including studies published after the year 2000, we have to say that our aim was to focus research on the twenty-first century, with the aim of analysing and including the latest research  With regard to the languages of publication, English was chosen because it is the language used by the scientific community, and Spanish and Portuguese because they are the languages we know best and are official in many countries |
| Given the fact that the articles included in the systematic review presented a high level of heterogeneity, what kind of precautions did the authors take to guarantee the interpretation of the results? | As the reviewer rightly points out, the meta-analyses performed showed a high degree of heterogeneity. This leads us to interpret with caution the results obtained in the meta-analyses carried out, and this has been stated in the manuscript. In this sense, this warning has been highlighted in the discussion and conclusions sections of the paper presented, and even in its limitations. In this way, the researchers aim to ensure that the interpretation of the results obtained is properly understood by the scientific community |
| Discussion  Line 347 and line 351 are similar, please rewritten | These lines have been rewritten |
| The authors exposed two main questions to answer through the present systematic review. What did they discover regarding the effectiveness of interventions towards developing respect of boys and girls during sport? | A paragraph has been introduced to clarify the results found on the effectiveness of interventions to develop respect for boys and girls during sport.  On the other hand, to answer the questions of the review and meta-analysis raised above, we have to say that, overall, the interventions carried out in the studies analyzed significantly promoted respect for opponents and social conventions. In terms of the characteristics of the interventions that led to an improvement in respect, the results showed that interventions based on models of sports education and personal and social responsibility achieved a significant increase in respect |
| The authors exposed the importance of the application model of Sport Education as well as the Personal and social Responsibility model or even the Fair Play Programme. Summing up, the authors revealed that three models will be important to promote and develop respect. But did they detect preferable situations for each one to be applied? | As the reviewer points out, all three models made significant improvements. The sports education model was used in secondary education, the personal and social responsibility model in secondary education and in a football club, while the fair play programme in primary education. In this respect, we have to say that, with the data provided by the studies analysed, no preferable situations were identified for the application of the different models  We believe that the question raised by the reviewer is interesting and relevant and that further research is needed to answer it. For this reason, we have added a future line of research to the article  In addition, in order to determine the most appropriate context for the implementation of a particular model, these models could be applied in different situations (primary education, secondary education, sports schools, sports clubs, etc.). This would allow us to test their effectiveness in developing respect, and to analyse in which contexts they work best |

**THANK YOU** for your comments and suggestions
