# Supplementary material for: The development of respect in young athletes: A systematic review and meta-analysis
Source: PLoS One. 2021 Jun 3;16(6):e0252643. doi: 10.1371/journal.pone.0252643 (PMC8174680; doi:10.1371/journal.pone.0252643)
Supplement: Attachment — Submitted filename: Response to Reviewers 3.docx [file pone.0252643.s004.docx]

**REBUTTAL LETTER**

Manuscript PONE-D-21-05952 [EMID:0e2927bc8a1101f0]

The development of respect in young athletes: A systematic review and meta-analysis

PLOS ONE

**Reviewer #5’s comments and suggestions for authors and details of the revisions and responses**

- 1) In the first paragraph of Results, lines 256-257—“93 were excluded because they were systemic review …”: in Figure it shows 96 instead of 93; “… and 383, after applying the exclusion. criteria”: it’s ambiguous, please revise.

We have made the changes indicated.

**THANK YOU** for your comments and suggestions.
